# Association of exposure to hydrocarbon air pollution with the incidence of atopic dermatitis in children

**DOI:** 10.1186/s13052-021-01157-7

**Published:** 2021-10-09

**Authors:** Chieh Wang, Chang-Ching Wei, Lei Wan, Cheng-Li Lin, Jeng-Dau Tsai

**Affiliations:** 1 Department of Chinese Medicine, China Medical University Hospital, Taichung, Taiwan; 2 Children’s Hospital, China Medical University Hospital, Taichung, Taiwan; 3 School of Medicine, China Medical University, Taichung, Taiwan; 4 School of Chinese Medicine, China Medical University, Taichung, Taiwan; 5 Management Office for Health Data, China Medical University Hospital, Taichung, Taiwan; 6 Institute of Biostatistics, China Medical University, Taichung, Taiwan; 7 School of Medicine, Chung Shan Medical University, Taichung, Taiwan; 8 Department of Pediatrics, Chung Shan Medical University Hospital, Taichung, Taiwan

**Keywords:** Air pollution, Atopic dermatitis, Children, Cohort study, Hydrocarbons, Non-methane hydrocarbon, Total hydrocarbon, Methane, Environmental pollutants

## Abstract

**Background:**

There is growing evidence suggesting that air pollution may act as an important environmental risk factor in the development and aggravation of childhood atopic dermatitis (AD).

**Methods:**

We collected data from the Taiwan National Health Insurance (NHI) research database and linked the data to the Taiwan Air Quality-Monitoring Database. From January 1, 2000 to December 31, 2012; children aged below 18 years were selected from the database and followed longitudinally until the diagnosis of AD, withdrawal from the NHI, or December 31, 2012. Children with missing data or those diagnosed with AD before enrolment in this study were excluded. We measured the incidence rate and hazard ratios (HRs) for AD and stratified them by quartiles (Q1–Q4) of air pollutant concentration. Multivariable Cox proportional hazards models were also applied by adjusting for age, sex, monthly income, and level of urbanization.

**Results:**

When compared with the concentrations of pollutants in the Q1 quartile, the adjusted HR for AD increased with an increase in the exposure concentrations of total hydrocarbons (THCs), non-methane hydrocarbons (NMHCs), and methane (CH_4_) from 1.65 (95% confidence interval [CI]: 1.47–1.84) to 10.6 (95% CI: 5.85–7.07), from 1.14 (95% CI: 1.06–1.24) to 2.47 (95% CI: 2.29–2.66), and from 1.70 (95% CI: 1.52–1.89) to 11.9 (95% CI: 10.8–13.1), respectively. Patients exposed to higher levels of THCs, NMHCs, and CH_4_ exhibited greater incidence rates of childhood AD.

**Conclusions:**

The present study demonstrated that exposure to higher concentrations of THCs, NMHCs, and CH_4_ were associated with an increased risk of childhood AD.

## Background

Atopic dermatitis (AD) is a common chronic relapsing inflammatory skin disease associated with intense itching and recurrent eczematous lesions [1]. Hanifin-Rajka major diagnostic criteria including pruritus, typical morphology, chronic or chronically relapsing dermatitis, and personal or family history of atopy have been used most frequently to diagnose AD in Taiwan [2]. In a process referred to as the “atopic march,” food allergy and AD are usually an early sign of other subsequent allergic disorders [3, 4]. Up to 80% of the children with AD eventually develop allergic rhinitis or asthma later in their childhood [5]. AD begins most commonly in the early childhood. Approximately 15–30% of the children and 10% of the adults are affected worldwide [6, 7]. In approximately 80% of the cases, childhood AD did not persist beyond 8 years of age and in less than 5% of the cases, it persisted into adulthood. Particularly, prolonged persistence of AD (more than 10 years) was associated with greater severity of the disease [8]. AD obviously influences patients’ quality of life and has financial implications. Itching and scratching are the two main symptoms that affect the quality of life in childhood AD. These symptoms affect the quality of sleep, thus requiring a treatment regime, affect the ability to participate in sporting activities, and result in social embarrassment [9]. The 2006 report from the American Academy of Dermatology, the most comprehensive contemporary research on the economic impact of AD, revealed that the total annual burden of AD was $4.228 billion. AD was associated with the fifth-highest overall treatment cost among all skin diseases in the US, placing a tremendous financial burden on the society [10]. Hence, it is critical to identify and control the risk factors in susceptible subjects for successful treatment and prevention of childhood AD.

Over the past 30 years, the worldwide prevalence of AD has increased considerably, particularly in industrialized countries [6]. Although both genetic and environmental factors are involved in the etiology of AD, the recent increase in the prevalence of is mainly attributed to environmental factors [11]. There is growing evidence suggesting that air pollution may act as an important environmental risk factor for the development and aggravation of childhood AD [12–15]. A variety of air pollutants such as particulate matter (PM); nitrogen oxide compounds (NO_x_); environmental tobacco smoke (ETS); traffic-related air pollution (TRAP) caused by pollutants such as PM, NO, NO_2_, SO_2,_ CO, CO_2_, O_3_; and volatile organic compounds (VOCs) have been mentioned as risk factors for the development or aggravation of AD [11]. Skin barrier dysfunction is considered the initial step in the development of AD. The skin barrier plays pivotal roles in immune surveillance and homeostasis and in preventing the penetration of irritants and allergens [6, 16, 17]. Air pollutants may induce oxidative stress in the skin, leading to skin barrier dysfunction or immune dysregulation [18, 19]. TRAP, especially O_3_, has been observed to alter the resident skin flora and cause predisposition to *S. aureus* colonization [18]. Other dust particles and diesel exhaust particulates have also been demonstrated to exert toxicological effects on human skin [19]. Further research is needed to determine the mechanism behind the role of air pollutants in AD.

Although several studies support the development or aggravation of childhood AD due to air pollutants, current evidence regarding the skin aspects of air pollution remains relatively scarce in contrast to that regarding airway diseases such as asthma [20]. Moreover, previous studies have limitations such as inaccurate study design and assessment and the presence of confounding variables such as obesity, genetics, and comorbidities. For example, several studies have considered a mixture of substances including ETS, VOCs, and NO_x_, representing a combined impact on human health. Selection bias was also observed due to potential misclassification in some cross-sectional studies, since the diagnosis of AD was not confirmed by a physician and was based simply on reports from the patients or their parents [11].

In the present study, we focused on the association between hydrocarbons and the development of childhood AD. Total hydrocarbons (THCs), which are organic chemical compounds consisting of non-methane hydrocarbons (NMHCs) and methane (CH_4_), are responsible for approximately 85% of the global energy consumption due to rapid *industrialization* and urbanization. It is unclear whether air pollutants released during combustion of hydrocarbons, particularly CH_4_, affect the body’s largest organ, the skin. Hence, we conducted this nationwide retrospective study using real-world data in Taiwan to evaluate the effect of exposure to these air pollutants on the risk of AD in children.

## Methods

### Data source

We conducted a retrospective cohort study using the Children’s File, a representative database including data from half of all children randomly selected from the registry of beneficiaries of the Taiwan National Health Insurance (NHI) Research Database (NHIRD) for the year 2000. The NHIRD was established in 1995 and covers more than 99% of the total population in Taiwan [21]. It contains all medical records including de-identified demographic information such as sex, birth dates, occupation of the beneficiaries, and place of residence and clinical information such as diagnostic codes based on the International Classification of Disease, 9th Revision, Clinical Modification [ICD-9-CM] [22]; health management; and treatment. Since all the research data were anonymized and encrypted to protect the individuals’ privacy, the requirement for consent was waived for this study. The study was approved by the Institute Review Board of China Medical University Hospital (CRREC-103-048) and the study procedures were in accordance with the principles of the Declaration of Helsinki.

### Study population, outcome of interest, endpoints, and confounding factors

From January 1, 2000 to December 31, 2012; We obtained data from children aged below 18 years. Candidates with missing data or those diagnosed with AD before enrolment in this study were excluded. The diagnosis of AD by a physician in Taiwan relies on clinical features listed in the Hanifin and Rajka diagnostic criteria and the American Academy of Dermatology Consensus Criteria [1, 2]. The AD diagnosis guideline established by the Taiwanese Dermatological Association committee (2015 and 2020) is also based on the aforementioned diagnostic criteria [1]. AD has chronic and relapsing characteristics. Hence, AD (the outcome of interest) was defined as at least three records of ICD-9-CM codes 691 (atopic dermatitis and related conditions) or 691.8 (other atopic dermatitis and related conditions) made by dermatologists or pediatricians in any diagnostic field during the inpatient or ambulatory claim process. All participants were followed up from baseline until the diagnosis of AD, withdrawal from the NHI, or December 31, 2012. The mean follow-up duration in AD patients was 6.50 years (standard deviation [SD]: 3.39 years). The confounding factors included age, sex, level of urbanization, and monthly income. The level of urbanization was defined based on the population density and was graded into four levels. Urbanization level was defined according to a National Health Research Institutes report [21]. City districts and townships where the subjects were registered for insurance purposes were grouped into seven urbanization levels based on population density (population/km^2^). Levels 1 and 7 referred to the most and the least urbanized areas, respectively. However, since very few patients were included in levels 5, 6, and 7; these three levels were combined with level 4. Monthly income was also classified into four groups: < 14,400 New Taiwan dollar (NT$); 14,400–18,300 NT$; 18,301–21,000 NT$; and ≥ 21,000 NT$.

### Exposure measurement

The Taiwan Air Quality Monitoring Network was established by the Taiwan Environmental Protection Administration (TEPA) in 1993 [23, 24]. It comprises of 74 monitoring stations around the island. The monitoring stations are fully automated and record daily readings of THCs, NMHCs, and CH_4_ using ultraviolet fluorescence. Air pollution data were extracted from all monitoring stations and averaged on each day. The databases of these air pollutants were obtained from the Taiwan Air Quality-Monitoring Database (TAQMD), released by the TEPA. We linked the NHIRD and the TAQMD data according to the residential areas of candidates and the location of air quality-monitoring stations. A residential area was defined based on the location of the clinic and the hospital that treated acute nasopharyngitis (common cold) (ICD-9-CM code 460). Since acute nasopharyngitis is a common health problem, patients tend to visit the local clinic or other medical institution nearest to their residential areas. The average daily concentrations of air pollutants were calculated by dividing the cumulative daily air pollutant concentration by the duration from enrolment in this study to the endpoint for each candidate. Air pollutant concentrations were categorized into four groups based on quartiles: Q1, Q2, Q3, and Q4. THC concentrations were categorized into Q1 (< 2.29 ppm), Q2 (2.29–2.40 ppm), Q3 (2.40–2.60 ppm), and Q4 (> 2.60 ppm). NMHC concentrations were categorized into Q1 (< 0.27 ppm), Q2 (0.27–0.35 ppm), Q3 (0.35–0.51 ppm), and Q4 (> 0.51 ppm). CH_4_ concentrations were categorized into Q1 (< 2.01 ppm), Q2 (2.01–2.06 ppm), Q3 (2.06–2.11 ppm), and Q4 (> 2.11 ppm).

### Statistical analysis

The demographic data in our study included age, sex, monthly income, level of residential urbanization, and daily average exposure to air pollutants. The chi-squared test was used to analyze the distributed difference among daily average concentrations for each air pollutant by quartile and urbanization. We used person-years as the denominator for estimating the incidence rate. The incidence rate of AD (per 1000 person-years) was calculated at four different air pollutant concentration levels. Cox proportional hazards regression models were applied to estimate the hazard ratios (HRs) and 95% confidence intervals (CIs) for AD in Q2–Q4 levels of air pollutant concentrations when compared with the Q1 concentrations. The multivariable model was adjusted for age, sex, monthly income, and urbanization level. We also utilized the Kaplan–Meier method to estimate the cumulative incidence of AD during the follow-up. The log-rank test was used to analyze the difference among air pollutant concentration levels. All data analyses were performed using SAS 9.3 (SAS Institute Inc., Cary, NC, USA) and SPSS 15.1 (SPSS Inc., Chicago, IL, USA). The significance level was set at *p* < 0.05 in all statistical tests.

## Results

Altogether, 7304 children (2.96%) were diagnosed with AD within the cohort of 246,844 children (between January 1, 2001 and December 31, 2012). The demographic data of the patients are presented in Table 1. The mean age of the patients was 6.50 years (SD: 3.39 years). The proportion of boys and girls was similar (51.6% vs. 48.4%). Most of the participants were from families belonging to the lowest monthly income category (83.4%) and resided in the most urbanized areas (33.2%).

**Table 1:** The demographic information of study population

*N* = 246,844		n	%
Gender	Boys	126,256	51.6
Age, years	mean, SD	6.50	3.39
	≦6	126,967	51.4
	7–12	101,653	41.2
	> 12	18,224	7.38
Monthly income (NTD)^a^	< 15,000	205,871	83.4
	15,000 − 19,999	30,871	12.5
	≥ 20,000	10,102	4.09
Urbanization level^b^	1 (highest)	81,827	33.2
	2	79,185	32.1
	3	47,013	19.1
	4 (lowest)	38,819	15.7
Exposure			
THC level (daily average)	mean, SD	2.43	0.23
NMHC level (daily average)	mean, SD	0.40	0.17
CH_4_ level (daily average)	mean, SD	2.03	0.13
Follow years	mean, SD	10.6	3.02
Outcome			
Atopic dermatitis		7304	2.96

*SD* standard deviation

^a^Monthly income, New Taiwan Dollar (NTD), 1 NTD is equal to 0.03 USD

^b^Urbanization level: The urbanization level was categorized by the population density of the residential area into 4 levels, with level 1 as the most urbanized and level 4 as the least urbanized. *THC* total hydrocarbons, *NMHCs* non-methane hydrocarbons, *CH*_4_ methane

We collected the data of participants under conditions of THC, NMHC, and CH_4_ exposure based on the location of the Taiwan Air Quality Monitoring station. Concentrations of each air pollutant were categorized by quartiles, ranging from Q1 (the lowest concentration) to Q4 (the highest concentration). Tables 2, 3 and 4 show the baseline characteristics of children exposed to four levels of THC, NMHC, and CH_4_ concentrations. Children with the highest THC, NMHC, and CH_4_ exposure concentrations lived in areas with higher urbanization.

**Table 2:** Baseline characteristics of participants exposed to quartile (Q1-Q4) daily average concentrations of total hydrocarbons (THC)

Variables		THC ***N*** = 246,844								
		Q1 *N* = 63,003		Q2 *N* = 60,660		Q3 *N* = 70,328		Q4 *N* = 52,853		*p*-value
		n	%	n	%	N	%	n	%	
Age	mean, SD^a^	5.58	2.61	5.66	2.76	7.15	3.71	7.71	3.83	< 0.001
Boys		32,841	52.1	31,400	51.8	36,293	51.6	26,722	50.6	< 0.001
Monthly income (NTD)^b^										< 0.001
	< 15,000	55,885	88.7	53,572	88.3	56,140	79.8	40,274	76.2	
	15,000 − 19,999	5794	9.20	5428	8.95	10,531	15.0	9118	17.3	
	≥ 20,000	1324	2.10	1660	2.74	3657	5.20	3461	6.55	
Urbanization level^c^										< 0.001
	1 (highest)	17,524	27.8	13,907	22.9	24,184	34.4	26,212	49.6	
	2	15,355	24.4	24,605	40.6	23,393	33.3	15,832	30.0	
	3	14,606	23.2	10,230	16.9	14,777	21.0	7400	14.0	
	4 (lowest)	15,518	24.6	11,918	19.7	7974	11.3	3409	6.45	
Outcome										
Atopic dermatitis		504	0.80	788	1.30	2863	4.07	3149	5.96	< 0.001

Chi-square test; ^a^One-way ANOVA; *SD* standard deviation

^b^Monthly income: New Taiwan Dollar (NTD), 1 NTD is equal to 0.03 USD

^c^Urbanization level: The urbanization level was categorized by the population density of the residential area into 4 levels, with level 1 as the most urbanized and level 4 as the least urbanized

**Table 3:** Baseline characteristics of participants exposed to quartile (Q1-Q4) daily average concentrations of non-methane hydrocarbons (NMHC)

Variables		NMHC ***N*** = 246,844								
		Q1 *N* = 55,312		Q2 *N* = 75,581		Q3 *N* = 54,687		Q4 *N* = 61,264		*p*-value
		n	%	n	%	N	%	n	%	
Age	mean, SD^a^	6.12	3.18	6.04	3.09	7.02	3.52	6.97	3.67	< 0.001
Boys		28,693	51.9	39,338	52.1	27,917	51.1	31,308	51.1	< 0.001
Monthly income (NTD)^b^										< 0.001
	< 15,000	47,529	85.9	64,814	85.8	44,185	80.8	49,343	80.5	
	15,000 − 19,999	5766	10.4	8467	11.2	7931	14.5	8707	14.2	
	≥ 20,000	2017	3.65	2300	3.04	2571	4.70	3214	5.25	
Urbanization level^c^										< 0.001
	1 (highest)	10,156	18.4	19,922	26.4	25,416	46.5	26,333	43.0	
	2	16,372	29.6	26,062	34.5	15,707	28.7	21,044	34.4	
	3	8878	16.1	19,178	25.4	9417	17.2	9540	15.6	
	4 (lowest)	19,906	36.0	10,419	13.8	4147	7.58	4347	7.10	
Outcome										
Atopic dermatitis		1046	1.89	1692	2.24	1878	3.43	2688	4.39	< 0.001

Chi-square test; ^a^One-way ANOVA; *SD* standard deviation

^b^Monthly income: New Taiwan Dollar (NTD), 1 NTD is equal to 0.03 USD

^c^Urbanization level: The urbanization level was categorized by the population density of the residential area into 4 levels, with level 1 as the most urbanized and level 4 as the least urbanized

**Table 4:** Baseline characteristics of participants exposed to quartile (Q1-Q4) daily average concentrations of methane (CH_4_)

Variables		CH_**4**_ ***N*** = 246,844								
		Q1 *N* = 57,832		Q2 *N* = 62,400		Q3 *N* = 64,035		Q4 *N* = 62,577		*p*-value
		n	%	n	%	N	%	n	%	
Age	mean, SD^a^	5.72	2.61	5.68	2.84	6.30	3.19	8.25	4.03	< 0.001
Boys		30,067	52.0	32,576	52.2	32,959	51.5	31,654	50.6	< 0.001
Monthly income (NTD)^b^										< 0.001
	< 15,000	50,495	87.3	55,406	88.8	54,061	84.4	45,909	73.4	
	15,000 − 19,999	6026	10.4	5210	8.35	7732	12.1	11,903	19.0	
	≥ 20,000	1311	2.27	1784	2.86	2242	3.50	4765	7.61	
Urbanization level^c^										< 0.001
	1 (highest)	17,455	30.2	19,681	31.5	23,868	37.3	20,823	33.3	
	2	14,939	25.8	22,318	35.8	22,219	34.7	19,709	31.5	
	3	14,376	24.9	11,388	18.3	10,443	16.3	10,806	17.3	
	4 (lowest)	11,062	19.1	9013	14.4	7505	11.7	11,239	18.0	
Outcome										
Atopic dermatitis		482	0.83	921	1.48	1713	2.68	4188	6.69	< 0.001

Chi-square test; ^a^One-way ANOVA; *SD* standard deviation

^b^Monthly income: New Taiwan Dollar (NTD), 1 NTD is equal to 0.03 USD

^c^Urbanization level: The urbanization level was categorized by the population density of the residential area into 4 levels, with level 1 as the most urbanized and level 4 as the least urbanized

Table 5 shows the increase in incidence rate of AD from 0.69 to 6.45, from 1.72 to 4.37, and from 0.73 to 7.74 per 1000 person-years with an increase in the THC, NMHC, and CH_4_ exposure concentrations, respectively. In the multivariable Cox proportional hazard regression, the adjusted HR for AD increased with an increase in the THC, NMHC, and CH_4_ exposure concentrations from 1.65 (95% CI: 1.47–1.84) to 10.6 (95% CI: 5.85–7.07), from 1.14 (95% CI: 1.06–1.24) to 2.47 (95% CI: 2.29–2.66), and from 1.70 (95% CI: 1.52–1.89) to 11.9 (95% CI: 10.8–13.1), respectively when compared with the corresponding exposure concentrations in the Q1 quartile (1.00) (Table 5).

**Table 5:** Comparisons of differences in atopic dermatitis incidences and associated HRs in participants exposed to quartile (Q1-Q4) daily average concentrations of air pollutants

	Pollutant levels	Event	PY	IR	cHR	95%CI	aHR	95%CI
THC								
Q1	63,003	504	729,958	0.69	Ref.		Ref.	
Q2	60,660	788	694,338	1.13	1.64	(1.47, 1.83)	1.65	(1.47, 1.84)
Q3	70,328	2863	695,742	4.12	5.72	(5.21, 6.29)	6.43	(5.85, 7.07)
Q4	52,853	3149	487,850	6.45	8.82	(8.03, 9.69)	10.6	(9.60, 11.6)
NMHC								
Q1	55,312	1046	606,958	1.72	Ref.		Ref.	
Q2	75,581	1692	834,767	2.03	1.18	(1.09, 1.27)	1.14	(1.06, 1.24)
Q3	54,687	1878	551,734	3.40	1.92	(1.78, 2.07)	1.93	(1.79, 2.09)
Q4	61,264	2688	614,430	4.37	2.48	(2.31, 2.66)	2.47	(2.29, 2.66)
CH_4_								
Q1	57,832	482	664,004	0.73	Ref.		Ref.	
Q2	62,400	921	713,125	1.29	1.79	(1.60, 1.99)	1.70	(1.52, 1.89)
Q3	64,035	1713	689,674	2.48	3.38	(3.05, 3.73)	3.32	(3.00, 3.67)
Q4	62,577	4188	541,086	7.74	9.99	(9.09, 11.0)	11.9	(10.8, 13.1)

*PY* person-years

*IR* Incidence rate, (per 1000 person-years)

*cHR* crude hazard ratio

*aHR* adjusted hazard ratio of a multivariate analysis, after adjustment for age, sex, monthly income, and urbanization level

*CI* vconfidence interval

*Ref.* reference group

*THC* total hydrocarbons, *NMHCs* non-methane hydrocarbons, *CH*_4_ methane

**Fig. 1:**
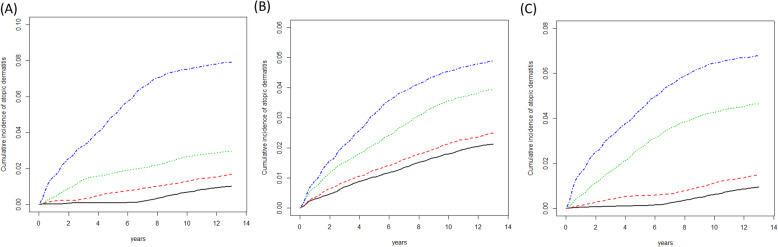
The cumulative incidence of atopic dermatitis in participants exposed to quartile daily average quartile concentrations of (**A**) total hydrocarbon (THC), (**B**) non-methane hydrocarbons (NMHC), and (**C**) methane (CH4). First quartile (solid line), first quartile to second quartile (dashed line), second quartile to third quartile (dotted line), ≧ third quartile concentration (dot dash line)

Figure 1 shows the Kaplan–Meier curves for the cumulative incidence separated by pollutant concentrations in each quartile (Q1, Q2, Q3, and Q4). During a follow-up of 12 years, the cumulative incidence rates of AD were lower among children living in areas with lower quartile concentrations of THCs, NMHCs, and CH_4_ than among those living in areas with higher quartile concentrations.

## Discussion

In the present population-based longitudinal study, we demonstrated that Taiwanese children exposed to higher concentrations of THCs, NMHCs, and CH_4_ were at an increased risk of developing AD regardless of adjustment for potential confounding factors such as age, sex, monthly income, and urbanization level. Our cohort study also revealed a clear dose-response relationship between air pollution and AD. The present study is distinctive in several respects. We assessed the real-world data from the Children’s File. Children are more susceptible than adults to the effects of air pollution, as their lungs and immune systems are still developing, they breathe faster than adults, and their respiratory tracts are more permeable [25]. AD diagnosis in our study was confirmed by a physician, minimizing the potential for selection bias. Our study might be one of the first to investigate the relationship between AD and CH_4_, an active greenhouse gas, to identify the dermatological effect of a single component.

Taiwan is located in East Asia, the most polluted region in the world. Currently, it is facing severe air pollution, especially in major urban areas, due to rapid increase in population and industrial development as well as transportation demands. While the number of children with AD continues to increase in both developed and developing countries, the prevalence of AD in Taiwan appears to have grown dramatically over recent decades [26]. According to the Taiwan National Study 2000 to 2007, the overall 8-year prevalence of AD is approximately 6.7% [27]. Due to such rapid growth in the number of AD cases with increased urbanization and industrialization, the role of environmental factors, especially airborne pollutants, has garnered increasing attention. Over the past 10 years, a number of studies have shown that TRAP and air pollutants such as PM, VOCs, and ETS are associated with the development and exacerbation of AD. Multiple comprehensive studies have been conducted in pediatric age group with a large dataset. In a French study that enrolled 4907 children who had resided at their current addresses for 3 years or longer, lifetime AD was significantly associated with 3-year averaged concentrations of PM_10_, NO_2_, NO_x_, and CO (adjusted odds ratios [ORs]: 1.13, 1.23, 1.06, and 1.08, respectively) [28]. In a prospective birth cohort study from Munich that included 2860 children aged 4 years, NO_2_ exposure (per 6.4 mg/m^3^) was associated with both physician-diagnosed AD and parental reports of AD symptoms (ORs: 1.18 and 1.11, respectively) [29]. In a cross-sectional study from Shanghai during 2011–2012 that enrolled 3358 preschool children, a positive correlation was observed between increased gestational and lifetime exposure to a mixture of SO_2_, NO_2_, and PM_10_ and childhood AD (ORs: 1.78 and 1.87, respectively) [30]. In 91,642 children from the US National Survey of Children’s Health, moderate to severe eczema was associated with elevated levels of NO_3_ and PM_2.5_ (ORs: 1.249 and 1.070, respectively) [31]. A few studies also revealed that prenatal exposure to VOCs and ETS are likely to induce a Th2-dominant immune status or the development of AD after birth [32–34]. In the present study, we observed that the adjusted HRs for AD increased with an increase in the exposure concentration of THCs (from 1.65 to 10.6), NMHCs (from 1.14 to 2.47), and CH_4_ (from 1.70 to 11.9) when compared with exposure to the corresponding concentrations in the Q1 quartile.

Rapid industrialization coupled with urbanization has led to accumulated global waste production due to the continuously increasing demand for energy. Hydrocarbons, which are organic chemical compounds consisting of hydrogen and carbon, form the basis of the majority of global energy production via fossil fuel combustion and evaporation of gasoline. Both NMHCs and CH_4_ are composed of THCs. Most of the hydrocarbons on earth are naturally derived from decomposition of organic matter in petroleum and are generated by human activity. NMHCs, often referred to as VOCs, are unstable forms of substances such as benzene and their derivatives.

A great number of animal and epidemiological studies have reported negative effects of VOCs on skin barrier function. A prospective study in Korea revealed that an increase of 1 ppb in outdoor benzene and total VOC concentrations was associated with a 27.38 and 25.86% increase in AD symptoms, respectively [14]. Kim et al. observed that exposure to airborne formaldehyde leads to an increase in transepidermal water loss and stratum corneum pH in healthy subjects as well as in AD patients [15]. In a rat AD model, Han et al. showed that formaldehyde exposure aggravated pruritus and skin inflammation. These results suggest that formaldehyde penetrated the injured skin barrier and exacerbated Th1 responses and serum IgE levels in AD rats [35]. In several previous studies, certain VOCs and polycyclic aromatic hydrocarbons have been proposed to activate the ligand-activated transcription factor AhR, leading to downstream activation of inflammation and itch mediators such as artemin [36, 37]. Adverse health effects of direct exposure to CH_4_, a nontoxic greenhouse gas, have been scarcely reported except suffocation due to high concentrations. The present study is the first one to suggest that CH_4_ exposure contributes to an increased risk of AD development. Rapid industrialization and urbanization contribute to increased CH_4_ production. Increasing urbanization has been accompanied by a rise in larger cities with increasing population densities. Densely populated areas aggravate the spread of contagious infectious diseases. Emerging infectious diseases may worse skin inflammation caused by AD [38]. Further studies are needed to confirm this hypothesis.

Although our study was a large-scale population-based cohort study, it has several limitations. Although AD is a complex and multifactorial disorder, we did not consider other environmental factors such as temperature, humidity, and ultraviolet light that might interact with airborne pollutants [39]. Other potential risk factors for AD such as atopic family history, dietary factors, pet and prenatal exposure, and even the severity of AD could not be estimated in the present study due to the lack of information in the Children’s File. The reported prevalence of AD was 7.2% in a previous study wherein 11,874 students from 14 schools in central Taiwan completed the International Study of Asthma and Allergies in Childhood questionnaire [40]. However, the present study revealed a prevalence of 2.96% during the study period. This finding implies that AD might have been underdiagnosed in the present study, particularly in those with mild or infrequent symptoms. This disparity in findings might be explained by the following factors. We defined AD as at least three medical records of ICD-9-CM codes 691 or 691.8 due to the chronic and relapsing nature of AD and to avoid overdiagnosis. Thus, although this approach reduced the risk of false positives, it might have led to a low prevalence of AD diagnosis during the study period. Patients with mild AD may not seek medical services or be coded for the clinical diagnosis of AD by a physician. Moreover, the selection of another ICD-9-CM code for the diagnosis of AD by a physician may also lead to underestimation of AD prevalence. Furthermore, due to the inclusion characteristics of our database, children enrolled during the study period who became older than 18 years of age in 2012 were also taken into account. Thus, the exposure measurement might have been calculated partially after the age of 18. Exposure to indoor air pollution was not investigated in our study. Hence, our results might not represent the overall effect of air pollution on AD [41].

## Conclusion

Our findings indicated that exposure to higher concentrations of THCs, NMHCs, and CH_4_ might lead to an increased risk of AD development. Further studies are needed to gain a better understanding of the role of air pollutants in the pathogenesis of AD.

## Data Availability

Data available on request due to privacy/ethical restrictions.

## Abbreviations

AD: Atopic dermatitis
CH_4_: Methane
CI: Confidence interval
ETS: Environmental tobacco smoke
HR: Hazard ratio
ICD-9-CM: International Classification of Disease, 9th Revision, Clinical Modification
NHI: National Health Insurance
NHIRD: National Health Insurance Research Database
NMHCs: Non-methane hydrocarbons
NO_x_: Nitrogen oxide compounds
NT$: New Taiwan dollar
OR: Odds ratio
PM: Particulate matter
SD: Standard deviation
TAQMD: Taiwan Air Quality-Monitoring Database
TEPA: Taiwan Environmental Protection Administration
THCs: Total hydrocarbons
TRAP: Traffic-related air pollution
VOC: Volatile organic compound
